# The ReceptIVFity cohort study protocol to validate the urogenital microbiome as predictor for IVF or IVF/ICSI outcome

**DOI:** 10.1186/s12978-018-0653-x

**Published:** 2018-12-07

**Authors:** Rivka Koedooder, Martin Singer, Sam Schoenmakers, Paul Hendrik Maria Savelkoul, Servaas Antonie Morré, Jonathan Dennis de Jonge, Linda Poort, Willem-Jan Simon Stephanus Cuypers, Andries Edward Budding, Joop Stephanus Elisabeth Laven, ReceptIVFity study group, K. Fleischer, B. J. Cohlen, C. B. Lambalk, J. M. J. S. Smeenk, N. G. M. Beckers, F. J. M. Broekmans, J. E. den Hartog

**Affiliations:** 1 Division of Reproductive Endocrinology and Infertility, Department of Obstetrics and Gynaecology, Erasmus University Medical Centre, Wytemaweg 80, 3015 CN Rotterdam, The Netherlands; 2 Laboratory of Immunogenetics, Department of Medical Microbiology and Infection Control, VU University Medical Centre, De Boelelaan 1108, 1081 HZ Amsterdam, the Netherlands; 3 Division Obstetrics, Department of Obstetrics and Gynaecology, Erasmus University Medical Centre, Wytemaweg 80, 3015 CN Rotterdam, The Netherlands; 4 Department of Medical Microbiology, School of Nutrition and Translational Research in Metabolism (NUTRIM), Maastricht University Medical Centre, P. Debyelaan 25, 6229 HX Maastricht, The Netherlands; 5 Institute of Public Health Genomics, Department of Genetics and Cell Biology, Research Institute GROW, Faculty of Health, Medicine & Life Sciences, University of Maastricht, Maastricht, The Netherlands; 6 ARTPred B.V., Kruisweg 647, 2131 NC Hoofddorp, The Netherlands; 7 IS-Diagnostics Ltd, spin-off at Department of Medical Microbiology and Infection Control, VU University Medical Centre, de Boelelaan 1108, 1081 HZ Amsterdam, The Netherlands; 8 Dutch Division, MVZ VivaNeo Kinderwunschzentrum Düsseldorf GmbH, Völklinger Straße 4, 40219 Düsseldorf, Germany

**Keywords:** Microbiota, Vaginal, Urinary, Implantation, Prediction

## Abstract

**Background:**

During the last decade, research has shown that besides the known predictive factors, such as duration of subfertility, a women’s age, the body mass index, also the microbiome might affect fertility. Micro-organisms together with their genetic information and the milieu in which they interact are called the microbiome. Studies have shown that the presence of certain microbiota during assisted reproductive technology (ART) has a positive impact on the outcome. However, the potential role of using the microbiome as a predictor for outcome of ART has not yet been investigated.

**Methods:**

In a prospective study, 300 women of reproductive age and with an indication for in-vitro Fertilization (IVF) with or without Intra Cytoplasmic Sperm Injection (ICSI) treatment will be included. Prior to the IVF or IVF-ICSI treatment, these women provided a midstream urine sample and a vaginal swab. The composition of the urinary and vaginal microbiome will be analysed with both Next Generation Sequencing and the IS-pro technique. The endpoints of the study are pregnancy achieved after fresh embryo transfer (ET) and within the subsequent year after inclusion. External validation of the findings will take place in an additional cohort of 50 women with an IVF or IVF-ICSI indication.

**Discussion:**

In the proposed study, the predictive accuracy of the composition of the urinary and vaginal microbiome for IVF or IVF-ICSI outcome will be only validated for fresh ET. Follow-up has to show whether the predictive accuracy will be similar during the consecutive frozen ET’s as part of the IVF or IVF-ICSI treatment or for subsequent stimulated or natural cycles. In addition, external validation will take place in another cohort and hospital. Predictive knowledge of the microbiome profile may enable couples to make a more substantiated decision on whether to continue treatment or not. Hence, the unnecessary physical and emotional burden of a failed IVF or IVF-ICSI treatment can be avoided.

**Trial registration:**

ISRCTN ISRCTN83157250. Registered 17 August 2018. Retrospectively registered.

## Plain English summary

In-vitro Fertilization (IVF) with or without Intra Cytoplasmic Sperm Injection (ICSI) is often the only option for subfertile couples that want to have children of their own. These treatments might cause considerable psycho-social stress and physical complaints. In this study we investigate whether we can predict IVF or IVF-ICSI outcome based on the vaginal bacteria which are carried by the women themselves.

Participants will be asked to self-collect a urine sample and a vaginal sample prior to the start of their IVF or IVF-ICSI treatment. The composition of the present bacteria will be analysed and linked to the fertility treatment or pregnancy outcome.

The objective of this study is assessment of the accuracy of a predictive algorithm based on the bacteria present in and on the woman herself. In addition, we will validate this predictive algorithm in an external population.

In conclusion: predictive knowledge based on the present bacteria may enable couples to make a more substantiated decision on whether to continue fertility (IVF or IVF-ICSI) treatment or not.

## Background

Assisted Reproductive Technologies (ART) such as in-vitro Fertilization (IVF) [1] with or without Intra Cytoplasmic Sperm Injection (ICSI) [2] are used to assist the 10–15% of couples affected by subfertility [3]. At present the pregnancy rate is around 25% for the first attempt (cycle) and cumulative ongoing pregnancy rates after additional cycles vary between 40 and 50% depending on the number of treatment cycles [4, 5]. Because of the psychosocial, physical and financial burden of ART, prediction of accurate outcome is needed. Current prediction models perform moderately well and are based upon pure clinical parameters such as age, number of previous failed IVF treatments, and the cause of subfertility [6].

However, at the department of Urology and the division of Reproductive Endocrinology and Infertility of the department of Obstetrics & Gynaecology both in the Erasmus University Medical Centre, a new predictive algorithm for the outcome of IVF or IVF-ICSI based on the microbial composition (population of different bacteria in a given sample) has been developed. The collection of microorganisms that live on or in the human body is known as our microbiota and its complete genetic profile as the microbiome. For this study, the urinary microbiome was determined in mid-stream urine sample. All urine samples were collected prior to the start of the actual IVF or IVF-ICSI treatment [7].

The predictive algorithm was developed in a pilot study comprising 42 women who were expected to start with either IVF or IVF-ICSI treatment and uses the percentages of the species *Lactobacillus*, *Staphylococcus* and *Escherichia coli* within the total microbiome of the sample. In the pilot study, the composition of the urinary microbiome prior to the start of the IVF or IVF-ICSI treatment was linked to the outcome of the ART treatment defined as ongoing pregnancy after one treatment cycle and pregnancy rates within 1 year after the initiation of the treatment. Cluster analysis and principal component analysis revealed that based on the microbiome composition it is possible to separate (by means of clustering) the women into two clusters: pregnant and non-pregnant women. After logistic regression, the bacterial species that dominated this prediction were identified and those were used to construct a predictive algorithm. In the pilot study, the test panel had a specificity of 96% and a sensitivity of 81%. Importantly, by adding the species *Bacillus RG4* the specificity improved up to 100%. The species found to be predictive has still to be confirmed in an independent, separate study. The crucial outcome of this predictive test is its specificity or better said, the prediction that the treatment will not result in pregnancy, since it could be used to select those women who should not be subjected to treatment because they will have a high chance not getting pregnant.

Hypotheses that might explain these finding is that the microbiome, acts as a sensor for the immunological tolerance that exist in secretory epithelia of a particular woman. Hence, it constitutes a proxy for endometrial receptivity which in turn depends on a similar immune response towards the developing embryo which is trying to implant. Another hypothesis is that the bacterial species included in the predictive algorithm possibly thrive the nutritional environment within epithelial secretions, which might be essential for initial survival of the embryo after transferral into the uterine cavity [8, 9].

In line with our finding that the genus *Lactobacillus* has an important role in reproductive health/outcome, are similar results of several published studies [10–20]. Recent publications show that women with infertility problems have a reduced number of *Lactobacillus* compared to healthy women [10–18]. Moreover, the presence of non-*Lactobacillus* dominated microbiota (e.g. presence of the genera *Gardnerella* and *Streptococcus*) seems to be associated with significant decreases in implantation, ongoing pregnancy and live birth rates [17] and clinical pregnancy rates [19, 20].

The large benefit of such a predictive test might have for patients undergoing IVF or IVF-ICSI treatment necessitates validation of these findings in a larger study with several independent ART clinics. Furthermore, to allow future clinical application of the algorithm, the Sanger sequencing technique used in the pilot study needs to be replaced by a quick and high-throughput method. Therefore, all collected microbiome samples will concomitantly be investigated with a Next Generation Sequencing (NGS) method as well as with the polymerase chain reaction (PCR) based IS-pro technique. These techniques will be used for both urine samples and vaginal samples, to investigate whether the urinary microbiome is affiliated from the vaginal microbiome. Here we describe the design of the ReceptIVFity cohort study.

## Methods

### Study aim

With ReceptIVFity, we aim to validate the algorithm in a larger cohort, and to develop a predictive test suitable for the use in daily practice. In this cohort study, the following aims will be addressed:

Primary Objective:

- To assess the specificity and sensitivity of the urinary and vaginal microbiome composition for the prediction of embryo implantation failure of a consecutive IVF or IVF-ICSI procedure.

Secondary Objective:

- To assess the specificity and sensitivity of the urinary and vaginal microbiome composition for the prediction of the cumulative outcome of 1 year of subsequently performed IVF or IVF-ICSI procedures.

### Study design and setting

The prospective study of the urogenital microbiome of subfertile women of reproductive age will be carried out in eight IVF centres in the Netherlands. The participating centres are: Erasmus University Medical Centre (UMC) (Rotterdam), Radboud UMC (Nijmegen), UMC Utrecht (Utrecht), VU University Medical Centre (Amsterdam), Isala Fertility Centre (Zwolle), Sint Elisabeth Hospital (Tilburg), VivaNeo Medical Centre Kinderwens (Leiderdorp), and Maastricht UMC+ (Maastricht). Inclusions will take place over the period from the 1st of June 2015 until the 31st March 2016. The external validation cohort will exist of women from the Dutch division of the MVZ VivaNeo Kinderwunschzentrum Düsseldorf GmbH (Germany) and inclusions will take place throughout 2018.

### Study population

Women who will visit the infertility outpatient clinics of participating hospitals and who are expected to undergo their first IVF or IVF-ICSI cycle within the next 2 months will be approached to participate in this study. Inclusion criteria to be fulfilled are: women aged between 20 and 44 years and a male partner. Those excluded from the study will be: women with an indication for emergency IVF because of cancer or other reasons, endometriosis American Fertility Score (AFS) III/IV and pre-treatment with a Gonadotrophin-releasing hormone (GnRH) analogue or those who use hormonal contraceptives within 3 months prior to the start of their IVF or IVF-ICSI intake. Those women who are using the oral contraceptive pill for the purpose of cycle timing prior to their treatment cycle will be eligible for this study. Women who have had a previous pregnancy or miscarriage in their medical history will also be excluded from participation. Pregnancy outcomes after the first fresh ET will be used as endpoint for this study. Ongoing pregnancy is defined as an intrauterine embryo/foetus with detection of cardiac activity on transvaginal ultrasound between 7 and 9 weeks of gestation.

### Study materials

A vaginal swab and a midstream urine sample before the start of the IVF or IVF-ICSI procedure will be self-collected. The swab and the urine sample have to be taken within the 2 months prior to the ET. A self-collecting method was chosen, because it is minimally invasive and therefore suitable for use in daily practice. Vaginal samples will be taken with FLOQSwabs™ (Copan Italia SpA, Brescia, Italy). The participants will be instructed to insert the swab 3–5 cm beyond the vaginal orifice, and move the swab around along the vaginal wall for 10–15 s. After this procedure the swabs will be immediately placed in Eppendorf tubes filled with reduced transport fluid (RTF) buffer (Microbiome Ltd., Amsterdam, the Netherlands). Until further processing, samples will be stored in a freezer at − 20 °C degrees.The urine sample collection will be obtained according to a standard ‘clean catch’ protocol, including washing hands thoroughly, cleaning the urinary opening with towelettes and collecting a midstream specimen in a sterile container. The urine sample will be stored at room temperature or in the refrigerator at 2–8 °C for a maximum of 2 hours until further processing. Further processing will consist of vortexing the urine sample and centrifuging 10 ml of the urine at 1500 relative centrifugal force (RCF) followed by resuspension in 1 ml of urine, which will be stored at -20 °C degrees until transport.Next, vaginal swabs and urine samples will be transported on dry ice by courier from the eight clinics to the microbiological laboratory.

### DNA isolation

DNA extraction of the vaginal swabs and urine samples will be performed with the Chemagen machine (Chemagen, Baesweiler, Germany) using the buccal swab extraction kit according to the manufacturer’s instructions. First, collected swabs and urine samples will be thawed and vortexed. 200 μl of sample will be incubated with 200 μl of Chemagen lysisbuffer and 10 μl Proteinase K (Qiagen, Hilden, Germany) at 56 °C while shaking at 500 rpm. DNA will be extracted using the protocol buccal Swab Prefilling. Finally, DNA will be eluted in 100 μl of Chemagen Elution buffer.

### Sequencing of the 16S ribosomal RNA

Picogreen dsDNA assay (Thermofisher, MA, USA) will be used for sample DNA concentration measurement. For sequencing, the V3-V4 region of the 16S rRNA gene region will be amplified using the individually distinguishable dual index primer sets. The rDNA amplification primers will be the universal primer set 319F/806R and they will be altered to also encode the Illumina sequencing primer and barcode labelling sequences. PCR will include 30 s at 98 °C, 30 cycles of 10 s at 98 °C, 15 s at 58 °C, and 15 s at 72 °C and three minutes at 72 °C. The AMPure XP magnetic bead assay (BeckmanCoulter Genomics, Danvers, MA, USA) will be used for purification of the amplified DNA. The following formula will be used for recalculation into nM and equalized to 12 nM:

$$ \left[\mathrm{nM}\ \mathrm{DNA}\right]=\frac{\left(\mathrm{DNA}\ \mathrm{concentration}\ \left(\frac{\mathrm{ng}}{\upmu \mathrm{l}}\right)x\ 1e6\ \left(\frac{\upmu \mathrm{l}}{\mathrm{L}}\right)\right)}{\Big(\mathrm{sample}\ \mathrm{fragment}\ \mathrm{size}\ \mathrm{in}\ \mathrm{bp}\ x\ \mathrm{656,4}\ \left(\mathrm{g}/\mathrm{mole}\right)} $$

If DNA concentrations fell below 12 nM, pooled DNA will be concentrated by vacuum evaporation.

### Next generation sequencing

NGS will be performed using a Miseq tabletop sequencer (Illumina, San Diego, CA, USA) by the Tumor Genome Analysis Core group of the Department of Pathology at the VU University Medical Centre in Amsterdam, The Netherlands. QIIME will be utilized to remove primer and index sequences, while paired end reads with a minimum overlap of six nucleotides and a minimum combined length of 400 nucleotides are assembled to produce identifiable sequences. The Usearch method will be utilized to produce operational taxonomic units (OTU) clusters. During this process, the sequences will be sorted on length and abundance of identical reads, will be checked for chimeric sequences and the sequence similarity threshold is set to 0.97 to denoise the data. The database described by Srinivasan et al. [21] will be used to assign sequences on a genus to species level by using the PyNAST method for sequence alignment and subsequently assignment using the RDP classifier method. The remaining sequences will be BLASTed, and will be included if the sequence can be identified at a genus or species level.

### Interspace profiling

Amplification of the intergenic spaces (IS) regions will be performed with the IS-pro assay, according to the protocol provided by the manufacturer (IS-Diagnostics, Amsterdam, the Netherlands). IS-pro is a eubacterial technique based on the detection and categorisation of the length of the 16S–23S rRNA gene IS region. The length of this IS region is specific for each bacterial species. Phylum-specific fluorescently labelled PCR primers will be used for taxonomic classification [22].

Briefly, the procedure consists of two separate standard PCRs: the first PCR mixture contains two different fluorescently labelled forward primers targeting different bacterial groups and three reverse primers providing universal coverage for those groups. The first forward primer is specific for the phyla *Firmicutes, Actinobacteria, Fusobacteria*, and *Verrucomicrobia* (FAFV), and the second labelled forward primer is specific for the phylum *Bacteroidetes*. A separate PCR mixture includes a labelled forward primer combined with seven reverse primers and is specific for the phylum *Proteobacteria*.

A GeneAmp 9700 PCR system (Applied Biosystems, Foster City, CA) will be used to perform the amplifications. After PCR, 5 μl of PCR product will be mixed with 20 μl of formamide and 0.2 μl of custom size marker (IS-Diagnostics, Amsterdam, The Netherlands). DNA fragment analysis will be performed on an ABI Prism 3500 genetic analyser (Applied Biosystems, Foster City, CA, USA). Data will be analysed with the IS-pro proprietary software suite (IS-Diagnostics, Amsterdam, The Netherlands), and the results will be presented as bacterial profiles. Automated species identification of IS-pro peaks will be done with the dedicated IS-pro software suite (IS-Diagnostics, Amsterdam, The Netherlands), in which peaks are linked to a database containing IS-profile information of > 500 microbial species. Peaks of < 500 relative fluorescence units (RFU) will be regarded as background noise and will be discarded from further analysis [22, 23].

### Microbiome analysis and algorithm building

The vaginal microbiome profile of each participant will be assigned to one of five community state types (CST) based on the dominant microbial species, as described by Ravel et al.^10^ Microbial communities in group I are dominated by *L. crispatus,* whereas group II, III, and V are dominated by respectively *L. gasseri, L. iners,* and *L. jensenii.* Group IV contains a heterogeneous group including species such as *Prevotella*, *Dialister*, *Atopobium*, *Gardnerella*, *Megasphaera*, *Peptoniphilus*, *Sneathia*, *Eggerthella*, *Aerococcus*, *Finegoldia*, and *Mobiluncus.*

The prediction model will be composed by analysis of the microbiological data will be performed in Spotfire 7.6 (TIBCO Spotfire Inc., Somerville, MA, USA). First, a distance matrix will be created on cosine distances of all possible sample pairs. Cosine distances are calculated with the following formula:

$$ \mathrm{dissimilarity}=1-\mathit{\cos}\left(\theta \right)=1-\frac{\sum_{i=1}^n{A}_i\times {B}_i}{\sqrt{\sum_{i=1}^n{\left({A}_i\right)}^2\times}\sqrt{\sum_{i=1}^n{\left({B}_i\right)}^2}} $$

The resulting data will be clustered with the unweighted pair-group method with arithmetic mean (UPGMA)4 and plotted as a heatmap.

To answer the questions of this study, an algorithm will be built based on the composition of the vaginal microbiome profile. First, the algorithm will be built by exploration of the pregnancy outcomes per CST. In a subsequent step, the data will be analysed for species content and microbial diversity per phylum. The formula will be validated for prediction of failure to become pregnant after (the first) fresh embryo transfer during the IVF or an IVF-ICSI treatment. Sensitivity will be calculated as true positive (TP) / (TP + false negative (FN)) and specificity as true negative (TN) / (TN + false positive (FP)). Predictive accuracy will be determined as the correlation between the predicted outcome and the actual outcome.

The vaginal samples that meet the prediction model for failure to become pregnant will be defined as ‘unfavourable microbiome profiles’. The vaginal samples that will not comply with the prediction model for failure to become pregnant will be defined as a ‘favourable microbiome profile’. Additionally, we will explore whether we can stratify these samples with a favourable microbiome profile into an average and a high chance to become pregnant based on additional bacterial species.

### Statistical analysis

Statistical analyses of the clinical data will be performed by using SPSS statistics version 24 (IBM corp, Amonk, NY, USA). We will examine two different prediction models. In the prediction model for failure to become pregnant, the primary outcome will be defined as ‘not pregnant’ after the first fresh ET during IVF or IVF-ICSI treatment. In the prediction model for success to become pregnant, the primary outcome will be defined as ‘pregnant’. Normality of data will be determined by using the Shapiro-Wilks normality test. Continuous, normally distributed variables will be presented as mean with standard deviation, and variables with a skewed distribution as median with the range. Categorical variables will be presented as count and proportions.

Differences between the groups of women who will become pregnant and those who will not will be compared using Chi-square test or Fisher’s exact test for the categorical data, as appropriate. The Independent Samples t-test and Mann-Whitney U test will be used for continuous data. Two-sided *P* values less than 0.05 will be considered statistically significant. Applying the same method, differences between women with a favourable microbiome profile and those with an unfavourable profile will be compared.

Multivariate analysis will be performed using logistic regression with a selection of covariates that are known predictors for pregnancy outcome; age, duration of infertility and body mass index (BMI).

## Discussion

In the ReceptIVFity cohort study, the specificity and sensitivity of the urogenital microbiome composition for the prediction of IVF or IVF-ICSI outcome will be determined in a cohort of women of reproductive age who will be expected to undergo their first IVF or IVF-ICSI cycle on short term. ReceptIVFity will investigate the role of a broad range of bacterial species and the influence of other clinical parameters in the success rate of IVF or an IVF-ICSI treatment. Insights in the microbial profile and their impact on the success rate may allow for a new strategy to decide whether to continue with treatment or not. The ultimate goal will be to develop a predictive algorithm that enables identification of the group of women with a low chance to become pregnant prior to the start of the IVF or IVF-ICSI treatment. Women with a low a priori chance to become pregnant might prefer to avoid the unnecessary physical and emotional burden of a IVF or IVF-ICSI treatment with a high change of failure.

### Strengths

The ReceptIVFity study will be the first cohort study in which the microbial composition will be used as predictor for IVF or IVF-ICSI outcome prior to the start of the treatment. As mentioned in the background section, several studies have found associations between the presence of microorganisms and reproductive outcomes, such as decreased implantation, pregnancy, ongoing pregnancy, and live birth rates. However, the clinical applicability of these findings has not yet been integrated into daily practice.

The ReceptIVFity cohort study consists of a large patient group/sample size and well-defined patients. A large sample is indicated, anticipating the fact patients can drop out for further treatment/follow up due to several reasons, such as poor response or total fertilisation failure. The included patients with embryo transfers will be used to investigate an association between microbial composition and IVF or IVF-ICSI outcome.

A prospective study design blinded for the test result, avoids bias to continue or not continue the treatment and allows for unbiased follow-up of the treatment outcomes.The 1 year follow-up is needed to examine the sustainability of the predicted outcome and will provide insight into the possibilities of becoming pregnant with subsequently performed IVF or IVF-ICSI procedures. Different techniques can be used to assess the microbiome and a high throughput technique is desirable for future application in daily practice. The prospective study validates the findings from the pilot study by the use of two different techniques. NGS that has become a gold standard for categorising bacteria or characterising microbial communities and the IS-pro technique which has the benefit of presenting results within 5 hours will be compared in this study. In addition, two sample sites (urine samples and vaginal swabs) will be compared with each other, both sites are easy to obtain by patients themselves. Developing a predictive test based on the urogenital microbiome composition will contribute to a personalised medicine approach in the future.

### Limitations

Because our study uses a well-defined study population, the results will be limited to the IVF or IVF-ICSI population. Whether these results also apply to a general population trying to establish a pregnancy and without ART cannot be extrapolated from these data.

In our study, we collect the samples only once and prior to the start of the first treatment cycle.

The success rate of IVF or IVF-ICSI treatment depends on multiple clinical parameters. Account must be taken for possible confounders in order to develop an independent test for prediction of IVF or IVF-ICSI outcome. Nevertheless, we will collect these clinical data (e.g. age, BMI, duration of infertility) and will correct for these possible confounders.

### In summary

In the future, microbiome profiling can be a routine diagnostic test prior to IVF or IVF-ICSI treatment if the negative predictive value is high enough to prevent patients a treatment with emotional and physical burden, that has low chance of success. With this cohort, we aim to contribute to better insight and validation of the urogenital microbiome as predictor for IVF or IVF-ICSI outcome. Finally, our ultimate goal is development of a diagnostic test that enables couples to make a more substantiated decision on whether to continue with treatment or not, based on their personal and individual microbiome profile.

## Abbreviations

AFS: American Fertility Score
ART: Assisted Reproductive Technologies
BMI: Body mass index
CST: Community state type
DNA: Deoxyribonucleic acid
ET: Embryo transfer
FAFV: *Fusobacteria* and *Verrucomicrobia*
FN: False negative
FP: False positive
GnRH: Gonadotrophin-releasing hormone
ICSI: Intra Cytoplasmic Sperm Injection
IS: Intergenic spaces
IVF: In-vitro Fertilization
NGS: Next generation sequencing
OTU: Operational Taxonomic Units
PCR: Polymerase chain reaction
RCF: Relative centrifugal force
RFU: Relative fluorescence units.
RTF: Reduced transport fluid.
TN: True negative
TP: True positive
UMC: University Medical Centre
UPGMA: Unweighted pair-group method with arithmetic mean
